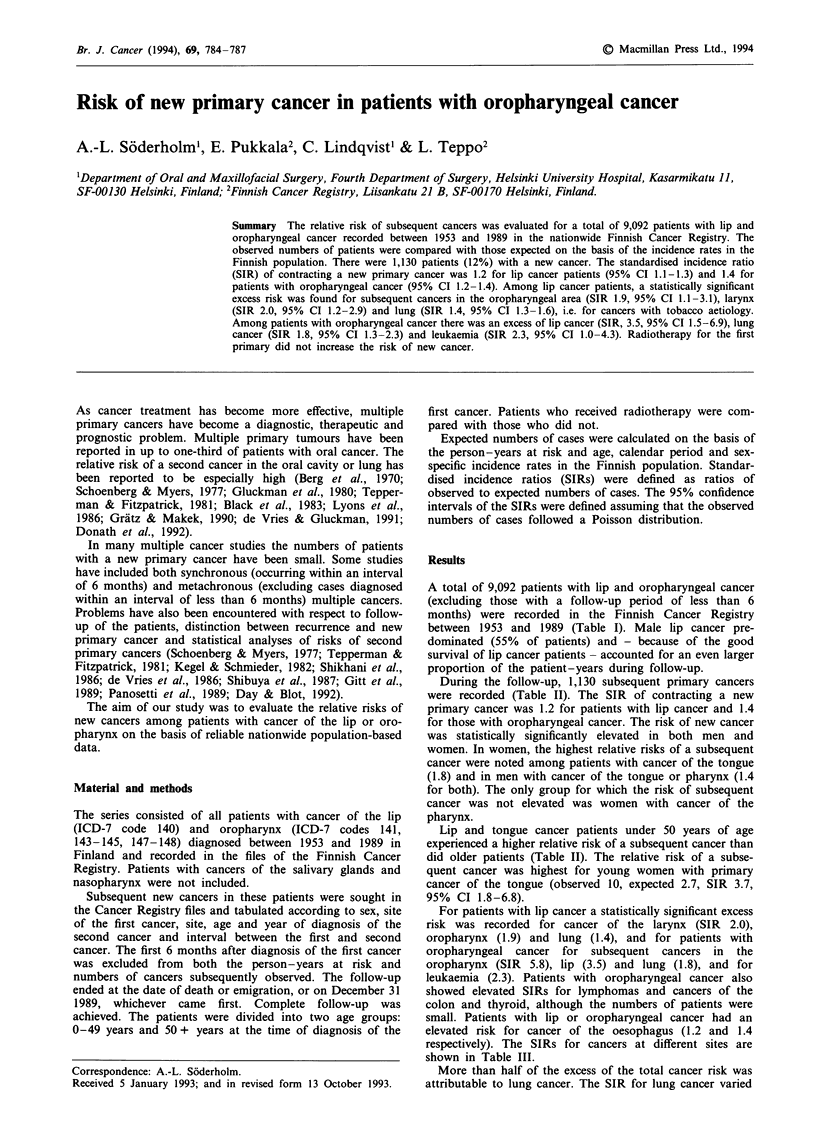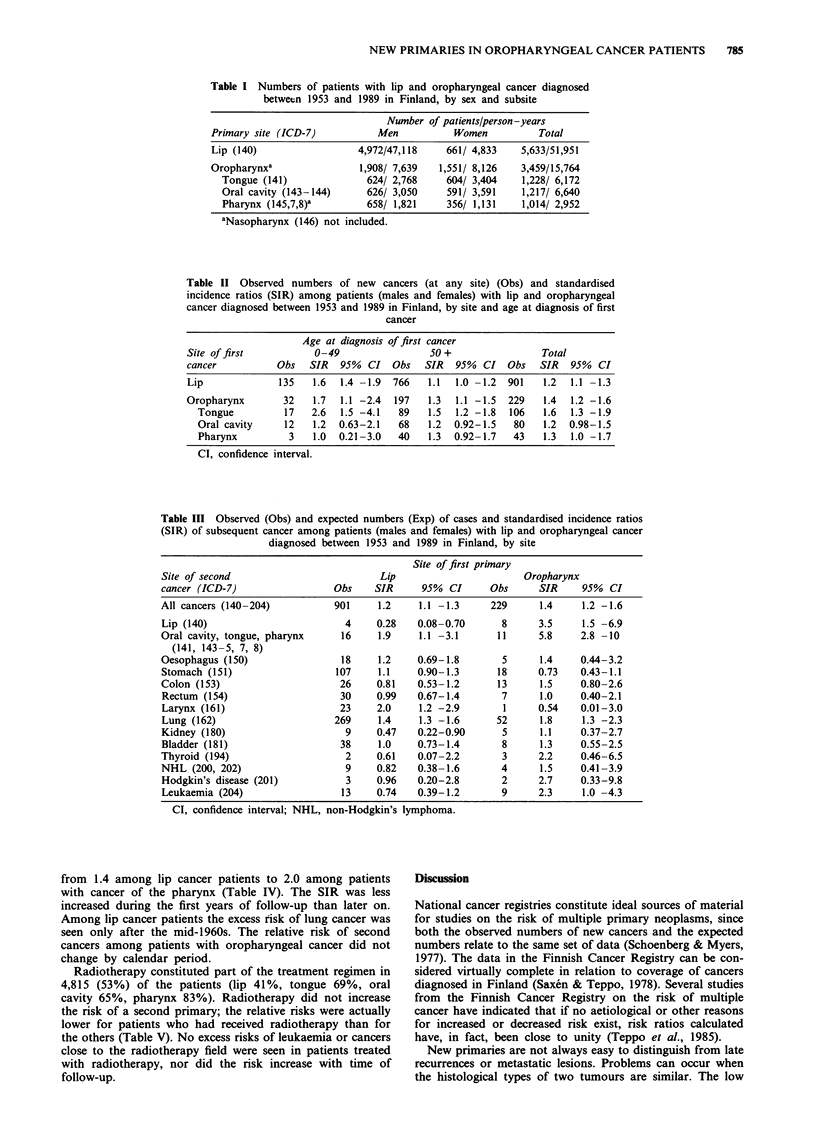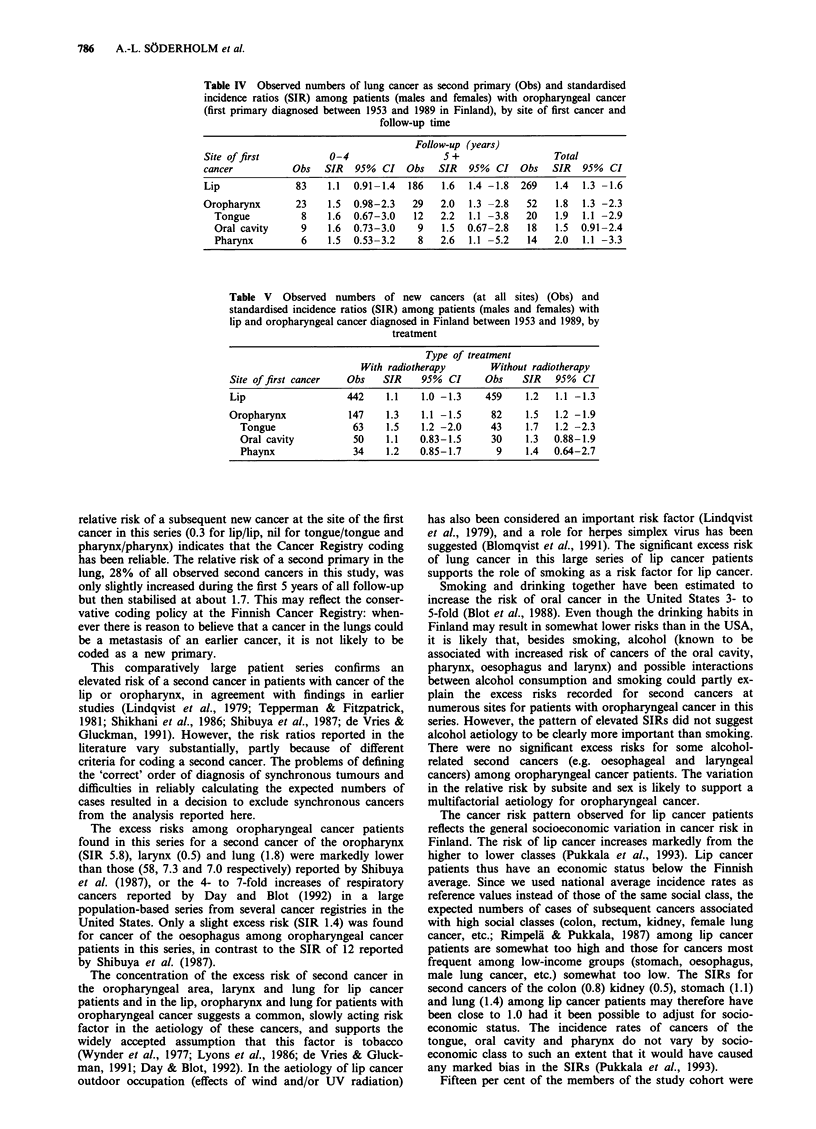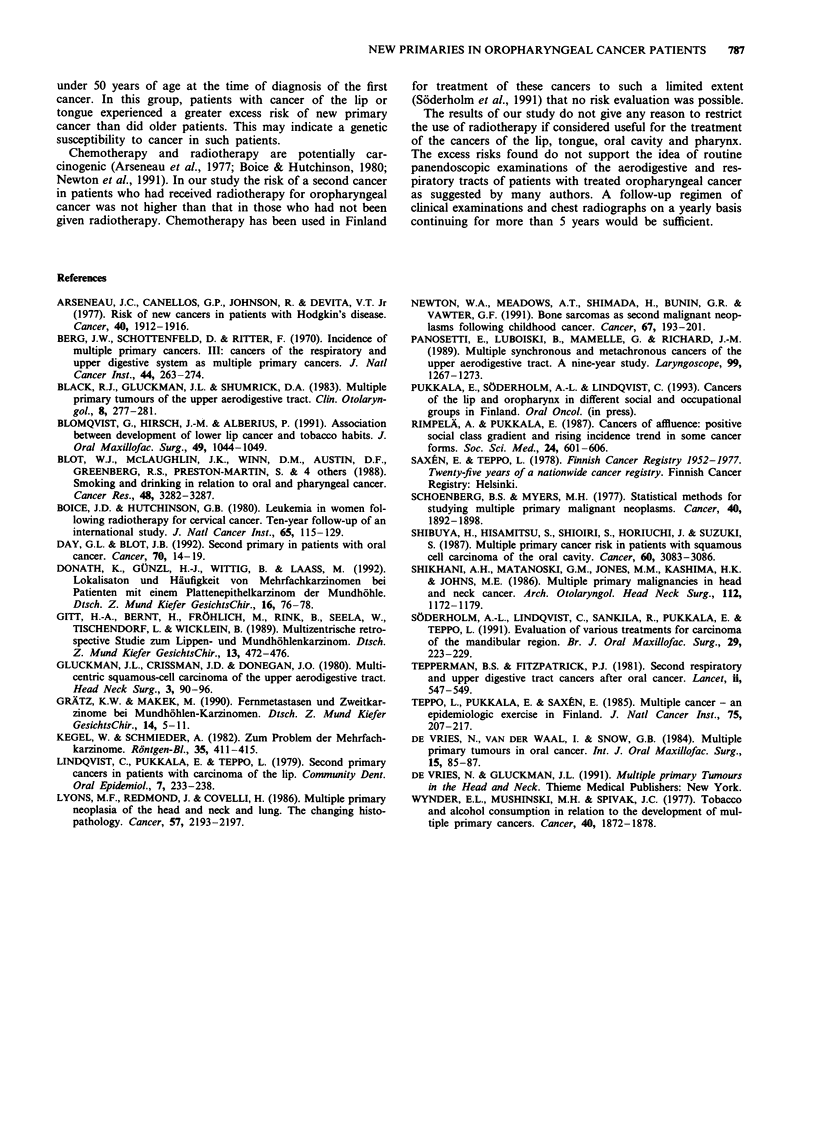# Risk of new primary cancer in patients with oropharyngeal cancer.

**DOI:** 10.1038/bjc.1994.148

**Published:** 1994-04

**Authors:** A. L. Söderholm, E. Pukkala, C. Lindqvist, L. Teppo

**Affiliations:** Department of Oral and Maxillofacial Surgery, Helsinki University Hospital, Finland.

## Abstract

The relative risk of subsequent cancers was evaluated for a total of 9,092 patients with lip and oropharyngeal cancer recorded between 1953 and 1989 in the nationwide Finnish Cancer Registry. The observed numbers of patients were compared with those expected on the basis of the incidence rates in the Finnish population. There were 1,130 patients (12%) with a new cancer. The standardised incidence ratio (SIR) of contracting a new primary cancer was 1.2 for lip cancer patients (95% CI 1.1-1.3) and 1.4 for patients with oropharyngeal cancer (95% CI 1.2-1.4). Among lip cancer patients, a statistically significant excess risk was found for subsequent cancers in the oropharyngeal area (SIR 1.9, 95% CI 1.1-3.1), larynx (SIR 2.0, 95% CI 1.2-2.9) and lung (SIR 1.4, 95% CI 1.3-1.6), i.e. for cancers with tobacco aetiology. Among patients with oropharyngeal cancer there was an excess of lip cancer (SIR, 3.5, 95% CI 1.5-6.9), lung cancer (SIR 1.8, 95% CI 1.3-2.3) and leukaemia (SIR 2.3, 95% CI 1.0-4.3). Radiotherapy for the first primary did not increase the risk of new cancer.


*Br. J. Cancer* (1994), **69**, 784–787 © Macmillan Press Ltd., 1994

### Risk of new primary cancer in patients with oropharyngeal cancer

A.-L. Söderholm[1], E. Pukkala[2], C. Lindqvist[1] & L. Teppo[2]

[1]*Department of Oral and Maxillofacial Surgery, Fourth Department of Surgery, Helsinki University Hospital, Kasarmikatu 11, SF-00130 Helsinki, Finland;* [2]*Finnish Cancer Registry, Liisankatu 21 B, SF-00170 Helsinki, Finland.*

**Summary** The relative risk of subsequent cancers was evaluated for a total of 9,092 patients with lip and oropharyngeal cancer recorded between 1953 and 1989 in the nationwide Finnish Cancer Registry. The observed numbers of patients were compared with those expected on the basis of the incidence rates in the Finnish population. There were 1,130 patients (12%) with a new cancer. The standardised incidence ratio (SIR) of contracting a new primary cancer was 1.2 for lip cancer patients (95% CI 1.1–1.3) and 1.4 for patients with oropharyngeal cancer (95% CI 1.2–1.4). Among lip cancer patients, a statistically significant excess risk was found for subsequent cancers in the oropharyngeal area (SIR 1.9, 95% CI 1.1–3.1), larynx (SIR 2.0, 95% CI 1.2–2.9) and lung (SIR 1.4, 95% CI 1.3–1.6), i.e. for cancers with tobacco aetiology. Among patients with oropharyngeal cancer there was an excess of lip cancer (SIR, 3.5, 95% CI 1.5–6.9), lung cancer (SIR 1.8, 95% CI 1.3–2.3) and leukaemia (SIR 2.3, 95% CI 1.0–4.3). Radiotherapy for the first primary did not increase the risk of new cancer.

As cancer treatment has become more effective, multiple primary cancers have become a diagnostic, therapeutic and prognostic problem. Multiple primary tumours have been reported in up to one-third of patients with oral cancer. The relative risk of a second cancer in the oral cavity or lung has been reported to be especially high (Berg *et al.*, 1970; Schoenberg & Myers, 1977; Gluckman *et al.*, 1980; Tepperman & Fitzpatrick, 1981; Black *et al.*, 1983; Lyons *et al.*, 1986; Grätz & Makek, 1990; de Vries & Gluckman, 1991; Donath *et al.*, 1992).

In many multiple cancer studies the numbers of patients with a new primary cancer have been small. Some studies have included both synchronous (occurring within an interval of 6 months) and metachronous (excluding cases diagnosed within an interval of less than 6 months) multiple cancers. Problems have also been encountered with respect to follow-up of the patients, distinction between recurrence and new primary cancer and statistical analyses of risks of second primary cancers (Schoenberg & Myers, 1977; Tepperman & Fitzpatrick, 1981; Kegel & Schmieder, 1982; Shikhani *et al.*, 1986; de Vries *et al.*, 1986; Shibuya *et al.*, 1987; Gitt *et al.*, 1989; Panosetti *et al.*, 1989; Day & Blot, 1992).

The aim of our study was to evaluate the relative risks of new cancers among patients with cancer of the lip or oropharynx on the basis of reliable nationwide population-based data.

### Material and methods

The series consisted of all patients with cancer of the lip (ICD-7 code 140) and oropharynx (ICD-7 codes 141, 143–145, 147–148) diagnosed between 1953 and 1989 in Finland and recorded in the files of the Finnish Cancer Registry. Patients with cancers of the salivary glands and nasopharynx were not included.

Subsequent new cancers in these patients were sought in the Cancer Registry files and tabulated according to sex, site of the first cancer, site, age and year of diagnosis of the second cancer and interval between the first and second cancer. The first 6 months after diagnosis of the first cancer was excluded from both the person–years at risk and numbers of cancers subsequently observed. The follow-up ended at the date of death or emigration, or on December 31 1989, whichever came first. Complete follow-up was achieved. The patients were divided into two age groups: 0–49 years and 50+ years at the time of diagnosis of the first cancer. Patients who received radiotherapy were compared with those who did not.

Expected numbers of cases were calculated on the basis of the person–years at risk and age, calendar period and sex-specific incidence rates in the Finnish population. Standardised incidence ratios (SIRs) were defined as ratios of observed to expected numbers of cases. The 95% confidence intervals of the SIRs were defined assuming that the observed numbers of cases followed a Poisson distribution.

### Results

A total of 9,092 patients with lip and oropharyngeal cancer (excluding those with a follow-up period of less than 6 months) were recorded in the Finnish Cancer Registry between 1953 and 1989 (Table I). Male lip cancer predominated (55% of patients) and – because of the good survival of lip cancer patients – accounted for an even larger proportion of the patient–years during follow-up.

During the follow-up, 1,130 subsequent primary cancers were recorded (Table II). The SIR of contracting a new primary cancer was 1.2 for patients with lip cancer and 1.4 for those with oropharyngeal cancer. The risk of new cancer was statistically significantly elevated in both men and women. In women, the highest relative risks of a subsequent cancer were noted among patients with cancer of the tongue (1.8) and in men with cancer of the tongue or pharynx (1.4 for both). The only group for which the risk of subsequent cancer was not elevated was women with cancer of the pharynx.

Lip and tongue cancer patients under 50 years of age experienced a higher relative risk of a subsequent cancer than did older patients (Table II). The relative risk of a subsequent cancer was highest for young women with primary cancer of the tongue (observed 10, expected 2.7, SIR 3.7, 95% CI 1.8–6.8).

For patients with lip cancer a statistically significant excess risk was recorded for cancer of the larynx (SIR 2.0), oropharynx (1.9) and lung (1.4), and for patients with oropharyngeal cancer for subsequent cancers in the oropharynx (SIR 5.8), lip (3.5) and lung (1.8), and for leukaemia (2.3). Patients with oropharyngeal cancer also showed elevated SIRs for lymphomas and cancers of the colon and thyroid, although the numbers of patients were small. Patients with lip or oropharyngeal cancer had an elevated risk for cancer of the oesophagus (1.2 and 1.4 respectively). The SIRs for cancers at different sites are shown in Table III.

More than half of the excess of the total cancer risk was attributable to lung cancer. The SIR for lung cancer varied

Correspondence: A.-L. Söderholm.
Received 5 January 1993; and in revised form 13 October 1993.

**Table I** Numbers of patients with lip and oropharyngeal cancer diagnosed between 1953 and 1989 in Finland, by sex and subsite

| *Primary site (ICD-7)* | *Number of patients/person-years* *Men* | *Women* | *Total* |
|---|---|---|---|
| Lip (140) | 4,972/47,118 | 661/ 4,833 | 5,633/51,951 |
| Oropharynx[a] | 1,908/ 7,639 | 1,551/ 8,126 | 3,459/15,764 |
| Tongue (141) | 624/ 2,768 | 604/ 3,404 | 1,228/ 6,172 |
| Oral cavity (143–144) | 626/ 3,050 | 591/ 3,591 | 1,217/ 6,640 |
| Pharynx (145,7,8)[a] | 658/ 1,821 | 356/ 1,131 | 1,014/ 2,952 |

[a]Nasopharynx (146) not included.

**Table II** Observed numbers of new cancers (at any site) (Obs) and standardised incidence ratios (SIR) among patients (males and females) with lip and oropharyngeal cancer diagnosed between 1953 and 1989 in Finland, by site and age at diagnosis of first cancer

| *Site of first cancer* | *Age at diagnosis of first cancer* *0–49* *Obs* | *SIR* | *95% CI* | *50+* *Obs* | *SIR* | *95% CI* | *Total* *Obs* | *SIR* | *95% CI* |
|---|---|---|---|---|---|---|---|---|---|
| Lip | 135 | 1.6 | 1.4 –1.9 | 766 | 1.1 | 1.0 –1.2 | 901 | 1.2 | 1.1 –1.3 |
| Oropharynx | 32 | 1.7 | 1.1 –2.4 | 197 | 1.3 | 1.1 –1.5 | 229 | 1.4 | 1.2 –1.6 |
| Tongue | 17 | 2.6 | 1.5 –4.1 | 89 | 1.5 | 1.2 –1.8 | 106 | 1.6 | 1.3 –1.9 |
| Oral cavity | 12 | 1.2 | 0.63–2.1 | 68 | 1.2 | 0.92–1.5 | 80 | 1.2 | 0.98–1.5 |
| Pharynx | 3 | 1.0 | 0.21–3.0 | 40 | 1.3 | 0.92–1.7 | 43 | 1.3 | 1.0 –1.7 |

CI, confidence interval.

**Table III** Observed (Obs) and expected numbers (Exp) of cases and standardised incidence ratios (SIR) of subsequent cancer among patients (males and females) with lip and oropharyngeal cancer diagnosed between 1953 and 1989 in Finland, by site

| *Site of second cancer (ICD-7)* | *Site of first primary* *Lip* *Obs* | *SIR* | *95% CI* | *Oropharynx* *Obs* | *SIR* | *95% CI* |
|---|---|---|---|---|---|---|
| All cancers (140–204) | 901 | 1.2 | 1.1 –1.3 | 229 | 1.4 | 1.2 –1.6 |
| Lip (140) | 4 | 0.28 | 0.08–0.70 | 8 | 3.5 | 1.5 –6.9 |
| Oral cavity, tongue, pharynx (141, 143–5, 7, 8) | 16 | 1.9 | 1.1 –3.1 | 11 | 5.8 | 2.8 –10 |
| Oesophagus (150) | 18 | 1.2 | 0.69–1.8 | 5 | 1.4 | 0.44–3.2 |
| Stomach (151) | 107 | 1.1 | 0.90–1.3 | 18 | 0.73 | 0.43–1.1 |
| Colon (153) | 26 | 0.81 | 0.53–1.2 | 13 | 1.5 | 0.80–2.6 |
| Rectum (154) | 30 | 0.99 | 0.67–1.4 | 7 | 1.0 | 0.40–2.1 |
| Larynx (161) | 23 | 2.0 | 1.2 –2.9 | 1 | 0.54 | 0.01–3.0 |
| Lung (162) | 269 | 1.4 | 1.3 –1.6 | 52 | 1.8 | 1.3 –2.3 |
| Kidney (180) | 9 | 0.47 | 0.22–0.90 | 5 | 1.1 | 0.37–2.7 |
| Bladder (181) | 38 | 1.0 | 0.73–1.4 | 8 | 1.3 | 0.55–2.5 |
| Thyroid (194) | 2 | 0.61 | 0.07–2.2 | 3 | 2.2 | 0.46–6.5 |
| NHL (200, 202) | 9 | 0.82 | 0.38–1.6 | 4 | 1.5 | 0.41–3.9 |
| Hodgkin's disease (201) | 3 | 0.96 | 0.20–2.8 | 2 | 2.7 | 0.33–9.8 |
| Leukaemia (204) | 13 | 0.74 | 0.39–1.2 | 9 | 2.3 | 1.0 –4.3 |

CI, confidence interval; NHL, non-Hodgkin's lymphoma.

from 1.4 among lip cancer patients to 2.0 among patients with cancer of the pharynx (Table IV). The SIR was less increased during the first years of follow-up than later on. Among lip cancer patients the excess risk of lung cancer was seen only after the mid-1960s. The relative risk of second cancers among patients with oropharyngeal cancer did not change by calendar period.

Radiotherapy constituted part of the treatment regimen in 4,815 (53%) of the patients (lip 41%, tongue 69%, oral cavity 65%, pharynx 83%). Radiotherapy did not increase the risk of a second primary; the relative risks were actually lower for patients who had received radiotherapy than for the others (Table V). No excess risks of leukaemia or cancers close to the radiotherapy field were seen in patients treated with radiotherapy, nor did the risk increase with time of follow-up.

### Discussion

National cancer registries constitute ideal sources of material for studies on the risk of multiple primary neoplasms, since both the observed numbers of new cancers and the expected numbers relate to the same set of data (Schoenberg & Myers, 1977). The data in the Finnish Cancer Registry can be considered virtually complete in relation to coverage of cancers diagnosed in Finland (Saxén & Teppo, 1978). Several studies from the Finnish Cancer Registry on the risk of multiple cancer have indicated that if no aetiological or other reasons for increased or decreased risk exist, risk ratios calculated have, in fact, been close to unity (Teppo *et al.*, 1985).

New primaries are not always easy to distinguish from late recurrences or metastatic lesions. Problems can occur when the histological types of two tumours are similar. The low

**Table IV** Observed numbers of lung cancer as second primary (Obs) and standardised incidence ratios (SIR) among patients (males and females) with oropharyngeal cancer (first primary diagnosed between 1953 and 1989 in Finland), by site of first cancer and follow-up time

| Site of first cancer | Follow-up (years) 0–4 Obs | SIR | 95% CI | 5+ Obs | SIR | 95% CI | Total Obs | SIR | 95% CI |
|---|---|---|---|---|---|---|---|---|---|
| Lip | 83 | 1.1 | 0.91–1.4 | 186 | 1.6 | 1.4 –1.8 | 269 | 1.4 | 1.3 –1.6 |
| Oropharynx | 23 | 1.5 | 0.98–2.3 | 29 | 2.0 | 1.3 –2.8 | 52 | 1.8 | 1.3 –2.3 |
| Tongue | 8 | 1.6 | 0.67–3.0 | 12 | 2.2 | 1.1 –3.8 | 20 | 1.9 | 1.1 –2.9 |
| Oral cavity | 9 | 1.6 | 0.73–3.0 | 9 | 1.5 | 0.67–2.8 | 18 | 1.5 | 0.91–2.4 |
| Pharynx | 6 | 1.5 | 0.53–3.2 | 8 | 2.6 | 1.1 –5.2 | 14 | 2.0 | 1.1 –3.3 |

**Table V** Observed numbers of new cancers (at all sites) (Obs) and standardised incidence ratios (SIR) among patients (males and females) with lip and oropharyngeal cancer diagnosed in Finland between 1953 and 1989, by treatment

| Site of first cancer | Type of treatment: With radiotherapy Obs | SIR | 95% CI | Without radiotherapy Obs | SIR | 95% CI |
|---|---|---|---|---|---|---|
| Lip | 442 | 1.1 | 1.0 –1.3 | 459 | 1.2 | 1.1 –1.3 |
| Oropharynx | 147 | 1.3 | 1.1 –1.5 | 82 | 1.5 | 1.2 –1.9 |
| Tongue | 63 | 1.5 | 1.2 –2.0 | 43 | 1.7 | 1.2 –2.3 |
| Oral cavity | 50 | 1.1 | 0.83–1.5 | 30 | 1.3 | 0.88–1.9 |
| Phaynx | 34 | 1.2 | 0.85–1.7 | 9 | 1.4 | 0.64–2.7 |

relative risk of a subsequent new cancer at the site of the first cancer in this series (0.3 for lip/lip, nil for tongue/tongue and pharynx/pharynx) indicates that the Cancer Registry coding has been reliable. The relative risk of a second primary in the lung, 28% of all observed second cancers in this study, was only slightly increased during the first 5 years of all follow-up but then stabilised at about 1.7. This may reflect the conservative coding policy at the Finnish Cancer Registry: whenever there is reason to believe that a cancer in the lungs could be a metastasis of an earlier cancer, it is not likely to be coded as a new primary.

This comparatively large patient series confirms an elevated risk of a second cancer in patients with cancer of the lip or oropharynx, in agreement with findings in earlier studies (Lindqvist *et al.*, 1979; Tepperman & Fitzpatrick, 1981; Shikhani *et al.*, 1986; Shibuya *et al.*, 1987; de Vries & Gluckman, 1991). However, the risk ratios reported in the literature vary substantially, partly because of different criteria for coding a second cancer. The problems of defining the 'correct' order of diagnosis of synchronous tumours and difficulties in reliably calculating the expected numbers of cases resulted in a decision to exclude synchronous cancers from the analysis reported here.

The excess risks among oropharyngeal cancer patients found in this series for a second cancer of the oropharynx (SIR 5.8), larynx (0.5) and lung (1.8) were markedly lower than those (58, 7.3 and 7.0 respectively) reported by Shibuya *et al.* (1987), or the 4- to 7-fold increases of respiratory cancers reported by Day and Blot (1992) in a large population-based series from several cancer registries in the United States. Only a slight excess risk (SIR 1.4) was found for cancer of the oesophagus among oropharyngeal cancer patients in this series, in contrast to the SIR of 12 reported by Shibuya *et al.* (1987).

The concentration of the excess risk of second cancer in the oropharyngeal area, larynx and lung for lip cancer patients and in the lip, oropharynx and lung for patients with oropharyngeal cancer suggests a common, slowly acting risk factor in the aetiology of these cancers, and supports the widely accepted assumption that this factor is tobacco (Wynder *et al.*, 1977; Lyons *et al.*, 1986; de Vries & Gluckman, 1991; Day & Blot, 1992). In the aetiology of lip cancer outdoor occupation (effects of wind and/or UV radiation) has also been considered an important risk factor (Lindqvist *et al.*, 1979), and a role for herpes simplex virus has been suggested (Blomqvist *et al.*, 1991). The significant excess risk of lung cancer in this large series of lip cancer patients supports the role of smoking as a risk factor for lip cancer.

Smoking and drinking together have been estimated to increase the risk of oral cancer in the United States 3- to 5-fold (Blot *et al.*, 1988). Even though the drinking habits in Finland may result in somewhat lower risks than in the USA, it is likely that, besides smoking, alcohol (known to be associated with increased risk of cancers of the oral cavity, pharynx, oesophagus and larynx) and possible interactions between alcohol consumption and smoking could partly explain the excess risks recorded for second cancers at numerous sites for patients with oropharyngeal cancer in this series. However, the pattern of elevated SIRs did not suggest alcohol aetiology to be clearly more important than smoking. There were no significant excess risks for some alcohol-related second cancers (e.g. oesophageal and laryngeal cancers) among oropharyngeal cancer patients. The variation in the relative risk by subsite and sex is likely to support a multifactorial aetiology for oropharyngeal cancer.

The cancer risk pattern observed for lip cancer patients reflects the general socioeconomic variation in cancer risk in Finland. The risk of lip cancer increases markedly from the higher to lower classes (Pukkala *et al.*, 1993). Lip cancer patients thus have an economic status below the Finnish average. Since we used national average incidence rates as reference values instead of those of the same social class, the expected numbers of cases of subsequent cancers associated with high social classes (colon, rectum, kidney, female lung cancer, etc.; Rimpelä & Pukkala, 1987) among lip cancer patients are somewhat too high and those for cancers most frequent among low-income groups (stomach, oesophagus, male lung cancer, etc.) somewhat too low. The SIRs for second cancers of the colon (0.8) kidney (0.5), stomach (1.1) and lung (1.4) among lip cancer patients may therefore have been close to 1.0 had it been possible to adjust for socioeconomic status. The incidence rates of cancers of the tongue, oral cavity and pharynx do not vary by socioeconomic class to such an extent that it would have caused any marked bias in the SIRs (Pukkala *et al.*, 1993).

Fifteen per cent of the members of the study cohort were under 50 years of age at the time of diagnosis of the first cancer. In this group, patients with cancer of the lip or tongue experienced a greater excess risk of new primary cancer than did older patients. This may indicate a genetic susceptibility to cancer in such patients.

Chemotherapy and radiotherapy are potentially carcinogenic (Arseneau *et al.*, 1977; Boice & Hutchinson, 1980; Newton *et al.*, 1991). In our study the risk of a second cancer in patients who had received radiotherapy for oropharyngeal cancer was not higher than that in those who had not been given radiotherapy. Chemotherapy has been used in Finland for treatment of these cancers to such a limited extent (Söderholm *et al.*, 1991) that no risk evaluation was possible.

The results of our study do not give any reason to restrict the use of radiotherapy if considered useful for the treatment of the cancers of the lip, tongue, oral cavity and pharynx. The excess risks found do not support the idea of routine panendoscopic examinations of the aerodigestive and respiratory tracts of patients with treated oropharyngeal cancer as suggested by many authors. A follow-up regimen of clinical examinations and chest radiographs on a yearly basis continuing for more than 5 years would be sufficient.

### References

ARSENEAU, J.C., CANELLOS, G.P., JOHNSON, R. & DEVITA, V.T. Jr (1977). Risk of new cancers in patients with Hodgkin's disease. *Cancer*, **40**, 1912–1916.

BERG, J.W., SCHOTTENFELD, D. & RITTER, F. (1970). Incidence of multiple primary cancers. III: cancers of the respiratory and upper digestive system as multiple primary cancers. *J. Natl Cancer Inst.*, **44**, 263–274.

BLACK, R.J., GLUCKMAN, J.L. & SHUMRICK, D.A. (1983). Multiple primary tumours of the upper aerodigestive tract. *Clin. Otolaryngol.*, **8**, 277–281.

BLOMQVIST, G., HIRSCH, J.-M. & ALBERIUS, P. (1991). Association between development of lower lip cancer and tobacco habits. *J. Oral Maxillofac. Surg.*, **49**, 1044–1049.

BLOT, W.J., MCLAUGHLIN, J.K., WINN, D.M., AUSTIN, D.F., GREENBERG, R.S., PRESTON-MARTIN, S. & 4 others (1988). Smoking and drinking in relation to oral and pharyngeal cancer. *Cancer Res.*, **48**, 3282–3287.

BOICE, J.D. & HUTCHINSON, G.B. (1980). Leukemia in women following radiotherapy for cervical cancer. Ten-year follow-up of an international study. *J. Natl Cancer Inst.*, **65**, 115–129.

DAY, G.L. & BLOT, J.B. (1992). Second primary in patients with oral cancer. *Cancer*, **70**, 14–19.

DONATH, K., GÜNZL, H.-J., WITTIG, B. & LAASS, M. (1992). Lokalisaton und Häufigkeit von Mehrfachkarzinomen bei Patienten mit einem Plattenepithelkarzinom der Mundhöhle. *Dtsch. Z. Mund Kiefer GesichtsChir.*, **16**, 76–78.

GITT, H.-A., BERNT, H., FRÖHLICH, M., RINK, B., SEELA, W., TISCHENDORF, L. & WICKLEIN, B. (1989). Multizentrische retrospective Studie zum Lippen- und Mundhöhlenkarzinom. *Dtsch. Z. Mund Kiefer GesichtsChir.*, **13**, 472–476.

GLUCKMAN, J.L., CRISSMAN, J.D. & DONEGAN, J.O. (1980). Multicentric squamous-cell carcinoma of the upper aerodigestive tract. *Head Neck Surg.*, **3**, 90–96.

GRÄTZ, K.W. & MAKEK, M. (1990). Fernmetastasen und Zweitkarzinome bei Mundhöhlen-Karzinomen. *Dtsch. Z. Mund Kiefer GesichtsChir.*, **14**, 5–11.

KEGEL, W. & SCHMIEDER, A. (1982). Zum Problem der Mehrfachkarzinome. *Röntgen-Bl.*, **35**, 411–415.

LINDQVIST, C., PUKKALA, E. & TEPPO, L. (1979). Second primary cancers in patients with carcinoma of the lip. *Community Dent. Oral Epidemiol.*, **7**, 233–238.

LYONS, M.F., REDMOND, J. & COVELLI, H. (1986). Multiple primary neoplasia of the head and neck and lung. The changing histopathology. *Cancer*, **57**, 2193–2197.

NEWTON, W.A., MEADOWS, A.T., SHIMADA, H., BUNIN, G.R. & VAWTER, G.F. (1991). Bone sarcomas as second malignant neoplasms following childhood cancer. *Cancer*, **67**, 193–201.

PANOSETTI, E., LUBOISKI, B., MAMELLE, G. & RICHARD, J.-M. (1989). Multiple synchronous and metachronous cancers of the upper aerodigestive tract. A nine-year study. *Laryngoscope*, **99**, 1267–1273.

PUKKALA, E., SÖDERHOLM, A.-L. & LINDQVIST, C. (1993). Cancers of the lip and oropharynx in different social and occupational groups in Finland. *Oral Oncol.* (in press).

RIMPELÄ, A. & PUKKALA, E. (1987). Cancers of affluence: positive social class gradient and rising incidence trend in some cancer forms. *Soc. Sci. Med.*, **24**, 601–606.

SAXÉN, E. & TEPPO, L. (1978). *Finnish Cancer Registry 1952–1977. Twenty-five years of a nationwide cancer registry.* Finnish Cancer Registry: Helsinki.

SCHOENBERG, B.S. & MYERS, M.H. (1977). Statistical methods for studying multiple primary malignant neoplasms. *Cancer*, **40**, 1892–1898.

SHIBUYA, H., HISAMITSU, S., SHIOIRI, S., HORIUCHI, J. & SUZUKI, S. (1987). Multiple primary cancer risk in patients with squamous cell carcinoma of the oral cavity. *Cancer*, **60**, 3083–3086.

SHIKHANI, A.H., MATANOSKI, G.M., JONES, M.M., KASHIMA, H.K. & JOHNS, M.E. (1986). Multiple primary malignancies in head and neck cancer. *Arch. Otolaryngol. Head Neck Surg.*, **112**, 1172–1179.

SÖDERHOLM, A.-L., LINDQVIST, C., SANKILA, R., PUKKALA, E. & TEPPO, L. (1991). Evaluation of various treatments for carcinoma of the mandibular region. *Br. J. Oral Maxillofac. Surg.*, **29**, 223–229.

TEPPERMAN, B.S. & FITZPATRICK, P.J. (1981). Second respiratory and upper digestive tract cancers after oral cancer. *Lancet*, **ii**, 547–549.

TEPPO, L., PUKKALA, E. & SAXÉN, E. (1985). Multiple cancer – an epidemiologic exercise in Finland. *J. Natl Cancer Inst.*, **75**, 207–217.

DE VRIES, N., VAN DER WAAL, I. & SNOW, G.B. (1984). Multiple primary tumours in oral cancer. *Int. J. Oral Maxillofac. Surg.*, **15**, 85–87.

DE VRIES, N. & GLUCKMAN, J.L. (1991). *Multiple primary Tumours in the Head and Neck.* Thieme Medical Publishers: New York.

WYNDER, E.L., MUSHINSKI, M.H. & SPIVAK, J.C. (1977). Tobacco and alcohol consumption in relation to the development of multiple primary cancers. *Cancer*, **40**, 1872–1878.